# Sex and gender differences in myocarditis and dilated cardiomyopathy: An update

**DOI:** 10.3389/fcvm.2023.1129348

**Published:** 2023-03-02

**Authors:** DeLisa Fairweather, Danielle J. Beetler, Nicolas Musigk, Bettina Heidecker, Melissa A. Lyle, Leslie T. Cooper, Katelyn A. Bruno

**Affiliations:** ^1^Department of Cardiovascular Medicine, Mayo Clinic, Jacksonville, FL, United States; ^2^Department of Environmental Health Sciences and Engineering, Johns Hopkins Bloomberg School of Public Health, Baltimore, MD, United States; ^3^Center for Clinical and Translational Science, Mayo Clinic, Rochester, MN, United States; ^4^Mayo Clinic Graduate School of Biomedical Sciences, Mayo Clinic, Jacksonville, FL, United States; ^5^Department of Cardiology, Charité – Universitätsmedizin Berlin, Corporate Member of Freie Universität Berlin and Humboldt-Universität zu Berlin, Berlin, Germany; ^6^Division of Cardiovascular Medicine, Department of Medicine, University of Florida, Gainesville, FL, United States

**Keywords:** sex differences, gender differences, genetics, epidemiology, pathogenesis, devices, therapy

## Abstract

In the past decade there has been a growing interest in understanding sex and gender differences in myocarditis and dilated cardiomyopathy (DCM), and the purpose of this review is to provide an update on this topic including epidemiology, pathogenesis and clinical presentation, diagnosis and management. Recently, many clinical studies have been conducted examining sex differences in myocarditis. Studies consistently report that myocarditis occurs more often in men than women with a sex ratio ranging from 1:2–4 female to male. Studies reveal that DCM also has a sex ratio of around 1:3 women to men and this is also true for familial/genetic forms of DCM. Animal models have demonstrated that DCM develops after myocarditis in susceptible mouse strains and evidence exists for this progress clinically as well. A consistent finding is that myocarditis occurs primarily in men under 50 years of age, but in women after age 50 or post-menopause. In contrast, DCM typically occurs after age 50, although the age that post-myocarditis DCM occurs has not been investigated. In a small study, more men with myocarditis presented with symptoms of chest pain while women presented with dyspnea. Men with myocarditis have been found to have higher levels of heart failure biomarkers soluble ST2, creatine kinase, myoglobin and T helper 17-associated cytokines while women develop a better regulatory immune response. Studies of the pathogenesis of disease have found that Toll-like receptor (TLR)2 and TLR4 signaling pathways play a central role in increasing inflammation during myocarditis and in promoting remodeling and fibrosis that leads to DCM, and all of these pathways are elevated in males. Management of myocarditis follows heart failure guidelines and there are currently no disease-specific therapies. Research on standard heart failure medications reveal important sex differences. Overall, many advances in our understanding of the effect of biologic sex on myocarditis and DCM have occurred over the past decade, but many gaps in our understanding remain. A better understanding of sex and gender effects are needed to develop disease-targeted and individualized medicine approaches in the future.

## 1. Introduction

Men have an increased incidence of most cardiovascular diseases (CVDs) including atherosclerosis, myocardial infarction, myocarditis, dilated cardiomyopathy (DCM) and heart failure (1, 2). In 2013 we reviewed the topic of sex differences in myocarditis and DCM (3). At that time the National Institutes of Health (NIH) had not updated its guidance for the inclusion of sex as a biological variable (SABV) in study design, data analysis and reporting of findings for NIH supported studies (4–6), and few studies in the literature focused on the topic. In that review, we called for greater translational research efforts in understanding the pathogenesis of sex differences in myocarditis and expressed the need to develop multicenter biobanks that could link specific phenotypes of samples with a special focus on including both sexes (3). Nearly a decade later, there is a growing interest in understanding sex and gender differences in myocarditis and DCM with many publications on the topic. However, many of the same needs still exist, as described in the Executive Summary from the 2022 National Heart, Lung and Blood Institute (NHLBI) of NIH workshop on sex and gender outcomes related to COVID-19 (7). The focus of this review is to provide an update on sex and gender differences in myocarditis and DCM including recent information on the epidemiology, pathogenesis of disease, and clinical presentation, diagnosis and management.

## 2. Definitions

First, sex and gender are not interchangeable terms. *Sex* refers to biological differences attributed to chromosomes, hormones, reproductive anatomy, gene expression, etc., and typically refers to a binary of male or female but can include intersex (5, 8, 9). *Gender*, on the other hand, is a social construct that is rooted in biology but affected by environment and experience. Gender is not a binary, but a broad spectrum where individuals may identify as cis or transgender, non-binary, gender-neutral, or in other ways as they define their own gender (8, 10, 11). Biological sex does not change over time, but gender varies in different cultures and with time. This review summarizes data from studies over the past decade which focused on cis-gendered populations, but few if any studies have examined the role of gender on myocarditis or DCM. Although we do not specifically report data on gender differences in this review of myocarditis and DCM because the research has not yet been conducted (i.e., studies designed to understand the effect of gender on disease outcomes), it is important in a discussion of sex differences to understand that there are also environmental and social interactions (gender differences) that affect sex differences outcomes both for animal studies and human data.

*Myocarditis* is defined by the World Health Organization (WHO) and the International Society and Federation of Cardiology (ISFC) as myocardial inflammation (12) that can cause loss of heart function including sudden cardiac death (13, 14), heart failure, and/or DCM (15, 16). Myocarditis encompasses a number of subtypes including lymphocytic myocarditis, the most common form of myocarditis and the main topic of this review, fulminant myocarditis, giant cell myocarditis, eosinophilic myocarditis and autoimmune myocarditis. These types are not necessarily distinct from each other–lymphocytic myocarditis can be autoimmune and giant cell and eosinophilic myocarditis are often indistinguishable clinically. Regarding some classifications, the presence of necrosis (12, 17–19) and/or viral genome (19) is required for a definitive myocarditis or viral myocarditis diagnosis. Scientific statements and position statements differ with respect to the diagnostic certainty of cardiac magnetic resonance imaging (cMRI) for myocarditis. Histologic confirmation by endomyocardial biopsy (EMB) or surgical tissue analysis is required for a definite diagnosis of myocarditis based on the European Society of Cardiology Working Group on Myocardial and Pericardial Diseases position statement (19, 20). In contrast, cMRI is considered sufficient for a definite diagnosis of myocarditis according to the American Heart Association (AHA) scientific statement and the Brighton Criteria for vaccine-associated myocarditis (21, 22). Endomyocardial biopsies are not common practice in the United States, and the AHA statement recommends viral genome analysis only in cases of diagnostic uncertainty where the biopsy findings will directly influence treatment such as in suspected giant cell myocarditis or sarcoidosis (23). In 2018, native T1 and T2 weighted parametric mapping by cMRI were added to a modified 2009 Lake Louise criteria (LLC) for the diagnosis of myocarditis (24).

*Dilated cardiomyopathy* is a morphological disease classified by dilation of the ventricle(s) and/or impairment of contractional function (25) in the absence of other cardiac disease, including coronary artery disease (CAD), hypertension, or congenital heart disease (26–28). The WHO definition of DCM adds that these characteristics that disrupt the form and function of the heart may lead to other serious conditions including arrhythmia or heart failure (12, 22). DCM is further classified as either genetic or non-genetic, with environmental factors contributing to the pathogenesis of disease (29–31). The functional impairment that is characteristic of DCM is progressive–as the dilated heart pumps harder to keep up with systolic demand this increases strain which increases dilation, so that the long term consequence is heart failure (16). Much like myocarditis, DCM can have a number of causes that lead to cardiac damage, remodeling, and progression to DCM (32). Myocardial inflammation can initiate remodeling pathways that replace the damaged myocardium with fibrosis that over time leads to dilatation, which has been demonstrated in viral and autoimmune animal models of myocarditis; thus, myocarditis is regarded as a significant cause of DCM (33). If DCM is not found to have a genetic or any other clearly identifiable cause it is termed *idiopathic DCM*, with this group constituting the largest category of DCM (34). Aside from animal models, several clinical studies have shown that myocarditis can progress to DCM, which is sometimes referred to as *inflammatory DCM* although the term inflammatory cardiomyopathy is sometimes also used to refer to acute myocarditis (35–38). It is quite possible that all types of DCM have myocardial inflammation that are recruited to the heart if for no other reason than to heal fibrotic scar tissue, which is observed in animal models of myocarditis that progress to DCM (35, 36, 39–41). However, biopsies are rarely performed in patients with DCM to confirm the presence of inflammation. Additionally, there have been studies of idiopathic DCM that find evidence of viruses and cardiac inflammation suggesting the possibility that DCM may have progressed from myocarditis (42).

As heart failure is an important clinical outcome of both myocarditis and DCM, it warrants definition. *Heart failure* results from the inability of the heart to meet the metabolic needs of the body at normal pressures (43). Heart failure is a spectrum currently classified by clinical and functional measures that can occur suddenly resulting in sudden cardiac death or gradually develop as occurs when myocarditis progresses to DCM, which is often termed *chronic heart failure*. Myocarditis is an important cause of *sudden cardiac death*, particularly if highly exertional exercise occurs about a week after symptoms of a viral infection (44, 45). Typically, patients have no signs or symptoms of heart concerns prior to exercise. New York Heart Association (NYHA) class categorizes the severity of heart failure based on clinical cardiovascular fitness. Cardiac systolic function is also stratified into three categories of left ventricular ejection fraction (LVEF) (46): heart failure with reduced ejection fraction (HFrEF) (LVEF <40%), heart failure with middle range ejection fraction (HFmEF), and heart failure with preserved ejection fraction (HFpEF) (LVEF >49%) (46). This developing terminology indicates the limitations in diagnosing heart failure in the cardiovascular field.

## 3. Sex differences in epidemiology

Historically the epidemiology of myocarditis has been based mainly on small, single-center studies. The latest Global Burden of Disease (GBD) statistics place the prevalence of myocarditis and cardiomyopathy worldwide at 10.2 to 105.6 per 100,000 (47, 48), with an annual occurrence estimated at around 1.8 million cases (47). One recent study in Sweden reported that the incidence of myocarditis rose from 6.3 to 8.6 per 100,000 from 2000 to 2014 (49). In 2019, GBD statistics reported a mortality rate in patients with myocarditis aged 35–39 of 4.4 per 100,000 in women and 6.1 per 100,000 in men, indicating that more men die of myocarditis than women worldwide (47). Past studies examining sex differences in patients with myocarditis reported only a slightly higher prevalence of myocarditis in males than females (sex ratio female to male of 1:1.5 to 1:1.7) (50–52). In the past decade many clinical studies have been conducted examining sex differences in myocarditis. Studies consistently report that myocarditis occurs more often in men than women with a sex ratio ranging from 1:2–4 female to male (Table 1). Additionally, studies have found that myocarditis occurs more often in men under 50 years of age but in women after age 50 or post-menopause (25, 53–58). Several large studies found that myocarditis was most prevalent in young adult males aged 16–20 (53, 58), but one study of sudden cardiac death corroborated from autopsy in 42 cases reported that myocarditis occurred most often in males aged 36–45 years (Table 1) (56). Thus, overall myocarditis occurs most often in young men under age 50 who have higher biomarker levels (e.g., troponin, sST2) and worse outcomes, including mortality, compared to women (Table 1). Myocarditis occurs most often in women after menopause, and a recent study found that women with autoimmune myocarditis (confirmed presence of heart autoantibodies and anti-nuclear autoantibodies) had worse outcomes compared to men (Table 1) (59).

**TABLE 1:** Studies of sex differences in myocarditis in the past decade.

Year	Country	*n*	Sex ratio (F:M)	Main findings	References
2013	Finland	3,198	1:3.3	*Myocarditis more common in men than women (77% men) (*p* < 0.0001) *Median age of patients 33 years *Males were significantly younger than females (*p* < 0.0001) *Myocarditis most prevalent in men 16–20 years of age with a gradual decline with age thereafter *Women highest level after menopause *Hospital admissions inverse logarithmic association with age	(53)
2017	Israel	200	1:2.6	*More men had myocarditis than women (76% men) *Men had higher peak troponin levels (*p* < 0.001) *Men were significantly younger than women (*p* < 0.001) *More men were hospitalized (*p* = 0.015) *Women had more chronic medical conditions	(54)
2019	Many countries	303	1:3.5	*More men had myocarditis (78%) *sST2, a biomarker of heart failure, was increased in men with myocarditis <50 years of age *sST2 levels correlated with NYHA class heart failure in men but not in women	(55)
2019	Denmark	42	1:2.2	*More men were found to have myocarditis at autopsy for SCD (69% men) (*p* = 0.02) *SCD was higher in males for all ages from 16 to 45 *Highest SCD-myocarditis incidence from autopsy observed in those aged 36–45 years	(56)
2019	USA	27,129	1:2	*More men were hospitalized for myocarditis (66% vs. 34%) *Hospitalized men were younger than women (*p* < 0.001) *In hospital complications (6.5% vs. 2.5%) and mortality (3.5% vs. 1.8%) were higher in women (*p* < 0.001)	(254)
2020	Switzerland	51	1:4.1	*More men had myocarditis than women (82%) *Sex differences in symptoms: men chest pain, women dyspnea *Myoglobin and creatine kinase higher in men (*p* = 0.04, *p* = 0.004, respectively)	(162)
2020	New Zealand	178	1:2.5	*More men had myocarditis (71% men) *Men were younger than women (36 vs. 53 years, *p* < 0.001) *ST-elevation on electrocardiogram more often in men (*p* = 0.01)	(57)
2021	Poland	19,978	1:2.9	*More men were hospitalized with suspected myocarditis than women (74%) *Myocarditis occurred more often in patients 16–20 years of age *The proportion of males were higher in all age groups except patients >70 years of age *In the last 10 years, the incidence of myocarditis increased, especially in males	(58)
2022	South Africa	82	1:1.9	*More men had myocarditis (66%)	(255)
2022	Sweden	8,679	1:3	*Incidence rose from 6.3 to 8.6 per 100,000 from 2000 to 2014, mostly in men <50 years of age	(49)
2022	Italy	466	1:2.1	*More men had myocarditis (68%) *Lower LVEF predicted worse outcomes *Women with autoimmune anti-heart antibodies and/or antinuclear antibodies had worse outcomes (11% reported another autoimmune disease besides myocarditis and 12% had giant cell and/or eosinophilic myocarditis- forms thought to occur more often in women)	(59)

F, female; LVEF, left ventricle ejection fraction; M, male; *n*, number; NYHA, New York Heart Association; SCD, sudden cardiac death; sST2, soluble ST2; USA, United States of America.

Clinical and animal studies have demonstrated that myocarditis can progress to DCM (37–40, 60, 61). DCM has an estimated prevalence of 1 in 250 to 500 people (62) and the incidence has increased over the past decade (47). The worldwide prevalence of cardiomyopathy/DCM is higher in men and increases with age (47). Women have been reported to have better long-term survival from DCM following myocarditis, with more men requiring heart transplants (37, 43). We recently reviewed sex differences in DCM and reported an average overall 1:2.5 female to male sex ratio in a meta-analysis of 31 studies from a search of around 1,200 studies in the literature (32). Of the 31 studies reported in the study, most did not analyze data by sex. The overall sex ratio from the meta-analysis was similar to the population-based study of DCM conducted in Olmsted County, Minnesota in 1989, using the Rochester Epidemiology Project, which reported an age-adjusted sex ratio for both incidence and prevalence of 1:3 female to male (63). Similar to myocarditis, recent studies examining sex differences in DCM report that DCM occurs more often in men (60–77%) and that men have lower LVEF, worse outcomes, and higher mortality compared to women (Table 2). However, in contrast to myocarditis, DCM occurs more often after age 60 in both men and women (Table 2). However, the age that DCM occurs post-myocarditis has not been specifically examined.

**TABLE 2:** Studies of sex differences in DCM in the past decade.

Year	Country	*n*	Sex ratio (F:M)	Main findings	References
2011	USA	373	1:1.7	*More men developed DCM after myocarditis (62%) *Men had a worse outcome than women	(37)
2013	Western Europe	269	1:1.2	*Men had worse cardiac function with LVEF <45% (*p* < 0.001) *Arrhythmias and end-stage heart failure occurred more often in men (*p* < 0.001, *p* = 0.006, respectively) *Mortality higher in men than women	(256)
2013	China	288	1:1.9	*Men had association between polymorphism in IL-17 rs763780 and increased DCM risk	(173)
2014	Germany	140	1:3.4	*More men had DCM (77%) *Increased mortality in women with congestive heart failure (*p* = 0.001) and RVEF <38% (*p* = 0.006) (average age 59 ± 13)	(257)
2015	China	1,142	1:2.7	*More men developed DCM (73%) *Increased risk of mortality in men past 60 years of age with DCM compared to young men, but not for elderly women	(258)
2018	UK	881	1:2	*More men developed DCM (67%) *Men had lower LVEF (*p* = 0.019) *All-cause mortality higher in men than women *All-cause mortality increases with age, especially in men after 60 years of age	(259)
2019	Kathmandu	65	1:1.6	*More males developed DCM (61%) *DCM occurred more often past age 60	(260)

DCM, dilated cardiomyopathy; F, female; LVEF, left ventricle ejection fraction; M, male; *n*, number; NYHA, New York Heart Association; RVEF, right ventricle ejection fraction; sST2, soluble ST2.

## 4. Sex differences in genetics

### 4.1. Sex differences in genetic myocarditis

Until recently there were almost no reports of genetic associations with myocarditis; however, a number of sporadic cases of genetic associations of myocarditis emerged in the literature in recent years (64–72). Recurrent cases of myocarditis and patients presenting with ventricular arrhythmia most strongly suggest genetics could be a factor, especially in children (73–76). This was confirmed in adults in a recent international study of 23 hospitals that compared 36 patients with myocarditis with desmosomal gene variants to those with myocarditis without variants. The study found that patients with variants were at increased risk of recurrent myocarditis and ventricular arrhythmias, and that more women were affected than men (*p* = 0.01) (75). Another recent study performed next generation DNA sequencing on 36 patients with biopsy-confirmed myocarditis and found that 31% had evidence of genetic variants that have been associated with cardiomyopathy including *Titin* (*TTN*) (*n* = 8), *Desmoplakin* (*DSP*) (*n* = 1), *Filamin C* (*FLNC*) (*n* = 1) and *RNA binding protein 20* (*RBM20*) (*n* = 1) (77). A large study performed genetic sequencing on patients with myocarditis from three different registries and identified that 19 of 117 patients (16%) had a genetic variant associated with cardiomyopathy or neuromuscular disorders compared to 34 of 468 controls (7%, *p* = 0.003) (78). Pediatric cases with variants occurred more often in females, but in adults with myocarditis variants were found more often in males (78). *TTN* mutations were the most commonly found mutations in this study, occurring in 6 out of 8 males. The recent consensus statement for genetic testing in patients with inherited CVD does not recommend genetic testing in patients with myocarditis (79), but evidence may be lacking simply because researchers have not looked for the relationship before now. These recent studies suggest that it may be warranted. Why would myocarditis that is associated with genetic variants display sex differences? One possible explanation is that physical damage to the heart that occurs from genetic causes (i.e., pathogenic variants in titin) could be aggravated by secondary factors such as damage from toxins (i.e., chemotherapy agents like doxorubicin), autoimmunity (i.e., immune complex deposition) and/or infections (i.e., coxsackievirus) leading to inflammation that is sex-specific (32). Thus, the genetic issue may remain functionally and/or symptomatically “hidden” until a second “hit” occurs that may drive inflammation and cardiomyopathy (32).

### 4.2. Sex differences in genetic DCM

Dilated cardiomyopathy is inherited in about 30–40% of all cases, which has been reviewed extensively previously (34, 62, 80–82). Most familial DCM has an autosomal dominant inheritance pattern, which is expected to affect men and women equally. However, other inheritance patterns have been identified, including autosomal recessive, X-linked, and mitochondrial, where X-linked and mitochondrial DCM occur more often in women (83). Importantly, large scale genome-wide association studies that examined the risk associated with particular genetic profiles have found significant sex differences, indicating the importance of analyzing genetic data by sex (84). The most common genes associated with cardiomyopathy/DCM are listed in Table 3 in descending order [see also (32)]. However, more than 40 nuclear encoded or mitochondrial genes have been associated with DCM and fall into four major categories: proteins forming the myocyte cytoskeleton, sarcomeric proteins, nuclear envelope proteins, and calcium homeostasis/mitochondrial function regulators (82).

**TABLE 3:** Sex differences in the most common familial DCM-linked genes in descending order.

Sex difference	Gene type	Gene	Protein	Function	References
↑♂	sarcomere	*TTN/TTNtv*	Titin	sarcomere structure	
↑♂ Arrhythmia	non-sarcomere	*LMNA/C*	Lamin A/C	nuclear membrane envelope	(82, 86, 90)
↑♂ Arrhythmia	sarcomere	*MYH7*	α-Myosin heavy chain	sarcomere structure	
Unknown	sarcomere	*MYH6*	β-Myosin heavy chain	sarcomere structure	
↑♂		*BAG3*	BAG family molecular chaperone regulator 3	chaperone-assisted selective autophagy	(161)
Unknown	sarcomere	*MYPN*	Myopalladin	Z-disc of sarcomere	
↑♀		*DSP*	Desmoplakin	desmosome	(86)
↑♂ Cardiac events	sarcomere	*FLNC*	Filamin C	Z-disc of sarcomere	
♂ Affected younger and ↑ severity	non-sarcomere	*RMB20*	RNA binding protein 20	spliceosome	(92, 261)
No sex difference	sarcomere	*TNNT2*	Cardiac troponin T	sarcomere structure	(86)
Unknown	non-sarcomere	*SCN5A*	Sodium channel protein 5 subunit α	ion channel	
No sex difference	sarcomere	*TNNC1*	Cardiac troponin C	sarcomere structure	(86)
Unknown	sarcomere	*TNNI3*	Cardiac troponin I	sarcomere structure	
No sex difference	sarcomere	*TMP1*	Tropomyosin α1 chain	sarcomere structure	(86)
↑♂	sarcomere	*MYBPC3*	Myosin binding protein 3	sarcomere structure	(82)
↑♀	non-sarcomere	*PLN*	Cardiac phospholamban		(82)

^a^DCM, dilated cardiomyopathy; ♀, female; ↑, increased; ♂, male.

As shown in Table 3, familial DCM typically occurs more often in males, with a reported female to male sex ratio of 1:2–3 (32, 43, 85, 86). Titin truncating variants (*TTNtv*) show higher penetrance and younger age at presentation in men, who have higher rates of atrial fibrillation and worse cardiac function than women with these variants (82, 87). *TTNtv* women are at increased risk of developing peripartum cardiomyopathy, suggesting a role for sex hormones in influencing gene expression (88). Women with *Lamin A/C* (*LMNA*) DCM were found to have 45% less risk for life-threatening arrhythmia than men (89). Male *LMNA* mutation carriers present clinical manifestations at a younger age than females (90). Twelve studies that provided data on the sex distribution of *LMNA* variants in DCM patients and 6 similar studies on *Myosin binding protein 3* (*MYBPC3*) variants found that 98 out of 152 patients with *LMNA* variants (69%) and 60 out of 76 patients with *MYBPC3* variants (79%) were male (82). In contrast, the male proportion was significantly lower in cardiac *Phospholamban* (*PLN*) mutation carriers with DCM (46%). *PLN* was the only mutation examined with a female to male ratio >1 (54% female) (82). In one multicenter study, there was a trend toward a lower risk of major cardiovascular events in women who had *Filamin C* (*FLNC*) genetic variants (91). Males with pathogenic variants in the gene for *RBM20* were both significantly younger and had lower ejection fraction at diagnosis than females (*p* < 0.01) (92). Additionally, 35% of affected males (*n* = 11 of 31) needed a cardiac transplant while none of the affected females (*n* = 22) were this severe (*p* < 0.001) (92). Thus, although sex differences are found in familial DCM, many environmental factors contribute to these differences including damage to the heart from infections (e.g., viruses), toxins (e.g., chemotherapy, alcohol), inflammation (e.g., viral or autoimmune myocarditis), sex differences that exist in the basic physiology of the heart, and social factors that contribute to gender differences.

## 5. Sex differences in cardiovascular physiology

First, it is important to realize that every cell has a “sex” based on their sex chromosomes and is impacted by sex hormones from *in utero* throughout the lifespan. This results in clear differences in cardiac physiology and gene profiles according to sex (93). Female hearts are on average 25% smaller than male hearts with a smaller ventricular mass and diameter, yet greater cardiomyocyte contractility and ejection fraction (2, 94–97). The number and size (hypertrophy) of cardiac cells differ by sex for all cell types including cardiac myocytes and fibroblasts–which make up the greatest mass of the heart–with 30% of men having greater hypertrophy than women (1, 98). The sex difference in cardiac size is most apparent during adulthood, with myocardial mass better preserved in women as they age (2, 99, 100). Women have smaller coronary vessels than men, (101) premenopausal women have lower blood pressure (102, 103) but a faster resting heart rate than men (104), and women have higher LVEF percentages compared with men (105, 106). Hearts from men and women respond similarly to exercise, which stimulates healthy cardiac enlargement in both sexes, but is more pronounced in females (1, 2).

Many of these sex-specific effects on cardiac function are mediated by sex hormone receptors that signal using both non-genomic (receptors expressed on the surface of cells) and classic genomic mechanisms (107–111). It is important to realize that cells from both men and women express both estrogen receptors (ERs) and androgen receptors (ARs), just in differing ratios. For example, women have higher levels of ERs in/on their arteries than men (108). 17β-Estradiol (E2) signaling through ERs has been shown to prevent cardiac hypertrophy, inhibit reactive oxygen species-induced cardiac damage, prevent apoptosis in cardiac myocytes, and oppose mechanisms that lead to cardiac remodeling and fibrosis (Table 4) (112). The cardioprotective benefit of estrogen in females rapidly declines during menopause (around age 50) when older males have higher levels of circulating estrogen than aging females (113, 114). Little data exists on the effects of cycling hormones or hormonal changes during pregnancy on normal cardiac physiology in women. Hormone receptors also mediate their effects through sex hormone-dependent regulation of miRNAs that are delivered by extracellular vesicles (109, 110, 115). Additionally, chromosomal genotype and epigenetic regulation can also drive sex differences (1). The role of gender on basic cardiac function is understudied with virtually nothing in the literature.

**TABLE 4:** Effects of sex hormones and/or sex differences in the basic physiology of the heart.

	Effect	References
E2	Increases ApoE levels	(262, 263)
E2, ♀	Supports antioxidant effects/reduces oxidative stress (decreasing oxidases, lipid peroxidation, and superoxide anion formation and increasing reactive oxygen scavenging enzymes)	(41, 137, 262–266)
E2, ♀	Stimulates autophagy	(137, 267, 268)
E2	Maintains cardiac energy metabolism and protects mitochondrial function	(269)
E2	Decreases glucose utilization	(270)
♀	Promotes gene expression toward heart development	(1, 271)
♀	Smaller left ventricular end diastolic diameter	(272)
♀	Smaller size especially pre-menopause (mass and output) but more efficient pump (larger contractility and ejection fraction, more dense cardiomyocytes)	(2, 94–97)
♀	Preserves myocardial mass during aging	(99, 100)
♀	Decreases coronary vessel size, lower blood pressure, higher heart rate	(2, 273–275)
♀	Stiffer myocardial wall	(2)
Te	Levels higher in male hearts	(276)
♂	Increases hypertrophy	(98, 277, 278)
♂	Increases apoptosis	(279, 280)
Te	Reduces antibody/autoantibody formation during immune response	(281–283)
Te	Decreases ventricular repolarization time	(284)
♂	Increases endothelial cells with overall gene expression bias toward angiogenesis	(1, 271)
♂	Increases androgen receptor expression on monocytes	(285–287)
♂	Increases left ventricular mass and heart size, especially during puberty	(98, 273–275)

ApoE, Apolipoprotein E; E2, estrogen/17β-estradiol; ♀, female; ♂, male; Te, testosterone.

## 6. Sex differences in the pathogenesis of myocarditis and DCM

Most of our understanding of the pathogenesis of myocarditis and its progression to DCM comes from animal models. Several animal models of viral myocarditis and autoimmune myocarditis exist [reviewed in 15, 39, 116–118]. The first investigators to study sex differences in myocarditis were Huber and Woodruff, who reported in 1981 that male BALB/c mice had worse myocarditis in response to coxsackievirus B3 (CVB3) infection than females (119). Dr. Huber continued to study sex differences in myocarditis for her entire career, creating much of the field’s foundational understanding of sex differences in myocarditis. Some important findings include that males have more cardiac viral replication than females, which can be increased in females by the addition of testosterone (120). Male BALB/c mice with CVB3 myocarditis have a greater T helper (Th)1 response while females have a greater Th2 response, which can be converted to a Th1 response by the addition of testosterone (121). The predominant immune cell response in the Huber model of CVB3 myocarditis are γδ T cells, which vary by sex with males having more Vγ4 while females have more Vγ1 T cells and B cells (122). Using C57BL/6J chromosome Y consomic mice, Huber et al. showed that the Y chromosome also influences sex differences in myocarditis, although sex hormones mediate the largest effect (123). Genes that are expressed on the X chromosome such as TLR7 and TLR8 have also been found to play roles in regulating the innate immune response to infection contributing to sex differences in certain autoimmune disease models (124, 125). Roberts et al. reported sex differences in Toll-like receptor (TLR)2 and TLR4 signaling in C57BL/6 mice with CVB3 myocarditis, where there was increased expression of TLR2 in females but TLR4 in males (126, 127). And importantly, ERα was found to protect female mice with CVB3 myocarditis by decreasing the Th1 response while increasing regulatory T cells (Treg) while ERβ had the opposite effect (128). However, sex differences in the inflammatory infiltrate differ between models of viral myocarditis. The CVB3 model used by Huber (and many other researchers) causes widespread cardiac apoptosis/necrosis with only a low level of cardiac inflammation (15%) while the majority (70%) of mice die by day 7 with few surviving to develop DCM (117, 118). Using this model, investigators recently showed that an X-linked gene *Midline 1*/*Md1* that regulates TRIM18 expression reduces type I interferon levels in male C57BL/6 mice in response to CVB3 infection altering survival and myocarditis (129, 130). However, the investigators did not examine whether this gene contributed to sex differences in the immune response. Future studies should examine whether X-linked genes such as Md1/TRIM18, TLR7 and TLR8 contribute to sex differences in the immune response to viral infection and myocarditis to better understand the role of the X chromosome in disease pathogenesis.

**FIGURE 1:**
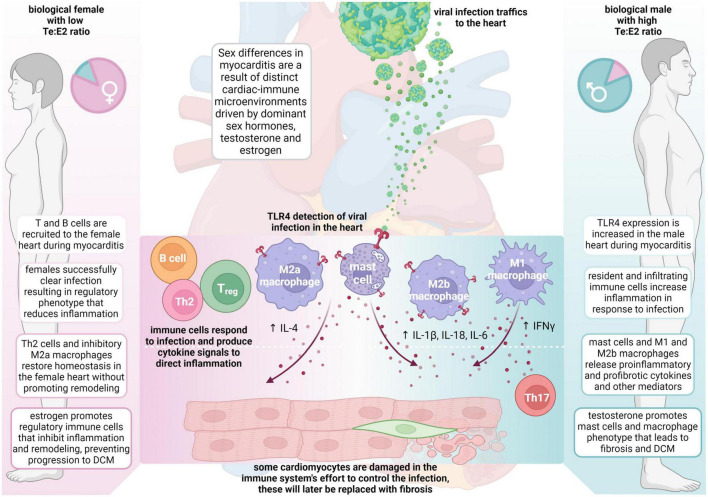
Sex differences in the pathogenesis of viral myocarditis and DCM. The summary of mechanisms of sex differences are primarily based on the Fairweather model of viral myocarditis (see text for full description). DCM, dilated cardiomyopathy; E2, estrogen/17β-estradiol; ♀, female; IFN, interferon; IL, interleukin; ♂, male; Te, testosterone; Th, T helper cells; TLR, Toll-like receptor.

Around 20 years after Dr. Huber started studying sex differences in CVB3 myocarditis, Dr. Fairweather developed a new model of CVB3 myocarditis based on the idea that myocarditis was an autoimmune disease, which used a mild CVB3 infection as the adjuvant combined with damaged heart protein as the antigen (15, 39, 116). In this model of myocarditis, male BALB/c mice develop worse myocarditis with a dominant immune infiltrate of CD11b/complement receptor 3 (CR3)+ immune cells including neutrophils, macrophages, mast cells and dendritic cells, but there are no sex differences in cardiac viral replication (Figure 1) (40, 131, 132). There is low apoptosis and relatively high inflammation (males average 60% vs. females 25% inflammation) and no deaths with all (100%) male and female BALB/c mice progressing to DCM (41). Males develop worse myocarditis and DCM and sequencing showed that the gene changes that lead to remodeling and fibrosis occur during acute myocarditis, and then time is all that is needed for collagen deposition and fibrosis to occur, with dilatation emerging around 30 days later (41, 133). This model is very similar to clinical lymphocytic myocarditis. Fairweather’s model showed that the dominant innate (first minutes and hours) and adaptive (during acute myocarditis at day 10 after infection) immune response in males is characterized by upregulation of complement and TLR4 on macrophages and mast cells (131, 132, 134, 135). IL-1β and IL-18 (interferon/IFNγ-inducing factor), which are produced from TLR4 signaling, lead to a dominant Th1 and M1 response in male BALB/c mice and cooperate with enzymes released from mast cells (i.e., α1-antichymotrypsin/serpin A 3n) to promote remodeling (3, 41, 133, 136, 137). Surprisingly, TLR4 was found to be present on alternatively activated M2 macrophages that resemble myeloid-derived suppressor cells with a profibrotic M2b phenotype in males, whereas females developed classic M2a macrophages with regulatory receptors like T cell immunoglobulin mucin (Tim)-3 (135). In contrast, a classic IFNγ and Th1 response protects against acute and chronic myocarditis/DCM in BALB/c mice by decreasing viral replication and preventing remodeling and fibrosis that leads to DCM (40, 134, 138). Fairweather showed that testosterone promotes, while 17β-estradiol inhibits myocarditis in BALB/c mice (55).

When C57BL/6 mice were used in this autoimmune viral model of CVB3 myocarditis, they developed a fulminant type of myocarditis (80% inflammation) characterized by a dominant classic Th1-type immune response but without deaths in wild type mice (139, 140). Fairweather found that a Th2-type immune response driven by activation of mast cells and a “mixed” Th1/Th2/M2b immune response in male BALB/c mice is required for the progression of myocarditis to DCM because of the critical role mast cells play in remodeling (Figure 1) (40, 139). In contrast, C57BL/6 mice, and other black background mice–which inherently have very few mast cells–do not progress to DCM after developing myocarditis (40, 141). Importantly, regardless of the organ, remodeling and fibrosis are known to require mast cells and are associated with a dominant Th2-type profibrotic immune response associated with IL-4, IL-33 and TGFβ1 (41, 133, 139). It is likely that IL-1β produced from mast cells and M2b macrophages in males increases IL-6, which in combination with TGFβ1, promotes a Th17 immune response that contributes to fibrosis and DCM (Figure 1) (142).

The effects of estrogen and/or female sex or testosterone and/or male sex on myocarditis and DCM from *in vivo* and *in vitro* studies of myocarditis/DCM are summarized in Tables 5, 6. Studies by other investigators using their own viral or autoimmune myocarditis animal models have found similar results to ours (Figure 1 and Tables 5, 6). Cardiac inflammation during myocarditis is strongly influenced by sex hormones on and in immune cells, as well as on and in cardiac tissue cells (3). ERα is primarily found in the uterus, liver, kidney, and heart (128). Sex hormone receptors are also located on and in many cells of the immune system including T cells, B cells, monocytes, macrophages, dendritic cells, and mast cells in humans and rodents (121). Importantly, only monocyte/macrophages and mast cells have *both* nuclear and membrane ERs and ARs (32, 121). ERα primarily controls E2 modulation of dendritic cell maturation, T cell cytokine production, and immunoglobulin responses (143–145). In contrast, signaling through ERβ up-regulates inducible nitric oxide synthase (iNOS) and nitric oxide generation with ERα suppressing this response (137, 146). Several important studies now show that when ERα and ERβ are co-expressed in the same cell, these receptors may exert opposing effects on gene expression and thus counterbalance each other (147–150).

**TABLE 5:** Effect of estrogen and/or female sex on an inflammatory response or myocarditis from animal models or tissue culture.

		Effect	References
myo	E2, ♀	Decreases myocarditis	(3, 41, 134, 135, 288–291)
myo	E2, ♀	Decreases TLR4 expression on innate immune cells (macrophages, mast cells)	(132, 266, 292–296)
myo	E2, ♀	Decreases TLR4-associated cytokines (TNF, IL-1, IL-6) and cardiac damage (CRP, CK)	(266, 272, 293–300)
myo	E2	Increases M2/M2a/myeloid-derived suppressor cell macrophages	(135, 289, 301–303)
myo	E2, ♀	Increases B cells and activates B cells to promote antibody and autoantibody response to infection or self-antigen	(135, 281–283, 304, 305)
myo	E2, ♀	Promotes Th2 response and regulatory T cells and increases IL-4 and IL-10 cytokines	(132, 134, 290)
myo	E2	Decreases IFNγ levels and Th1 cells after infection by inhibiting Tbet	(306–309)
myo	♀	Protection from oxidative stress after proinflammatory activation	(310)
DCM	♀	Female BALB/c mice develop less DCM after myocarditis with better LVEF	(41)
DCM	E2, ♀	Prevents cardiac hypertrophy and fibrosis as fibroblasts express fewer profibrotic factors	(1, 311–313)

CK, creatine kinase; CRP, C-reactive protein; DCM, dilated cardiomyopathy; E2, 17β1-estradiol; ♀, female; IL, interleukin; IFNγ, interferon gamma; LVEF, left ventricle ejection fraction; myo, myocarditis; Tbet, Tbx21; Th, T helper; TLR, Toll-like receptor; TNF, tumor necrosis factor.

**TABLE 6:** Effect of testosterone and/or male sex on an inflammatory response or myocarditis from animal models or tissue culture.

		Effect	References
Myo	Te, ♂	Increases myocarditis	(41, 132, 288, 314)
Myo	Te, ♂	Promotes inflammation through innate immune cells (mast cells, natural killer cells) and cytokines i.e., IFNγ)	(132, 288, 313, 315)
Myo	Te, ♂	Increases IL-18-induced Th1 response through TLR4+ mast cells and macrophages rather than classic IL-12/STAT4 pathway	(41, 132, 134, 135, 290, 302, 316–319)
Myo	Te, ♂	Increases leukocytes in the heart during myocarditis	(132, 288)
Myo	Te, ♂	Increases CD11b+ TSPO+ cells in the heart during myocarditis	(288)
Myo	♂	Increases expression of P450 oxidoreductase during myocarditis	(41)
DCM	Te, ♂	Increases progression to DCM after myocarditis with reduced LVEF	(41)
DCM	Te, ♂	Increases cardiac fibrosis after damage and/or infection	(1, 41, 251, 320–322)
DCM	♂	Th2-type immune response required for progression to DCM after myocarditis in males	(41, 133, 139–141)
DCM	♂	Increases proinflammatory phenotype of aged mice with DCM	(310, 323, 324)

DCM, dilated cardiomyopathy; IL, interleukin; ♂, male; myo, myocarditis; Th, T helper; Te, testosterone.

In general, estrogen has been found to increase immunoglobulin synthesis and inhibit B cell apoptosis resulting in increased antibody and autoantibody levels in females (151, 152), suppress both T and B cell lymphopoiesis (153), enhance dendritic cell differentiation and antigen presentation (154), suppress TNFα and IL-6 levels, (155, 156) increase IL-4 and IFNγ production (143, 157), and promote FoxP3+ T regulatory cell development. (32, 128, 158, 159).

During CVB3 myocarditis in the autoimmune CVB3 model, females generate a robust immune response to infection, but they regulate or inhibit the inflammatory response very well after clearing the viral infection by upregulating almost every regulatory immune feature, including antibodies, CR1, Tim-3, CTLA4, Treg, IL-10, anti-inflammatory and anti-fibrotic M2a-type macrophages, and so forth. In contrast, males develop a robust proinflammatory immune response but tend not to regulate it well, instead promoting mast cell and M2b cell responses that are both proinflammatory and profibrotic (Figure 1).

## 7. Sex and gender differences in clinical presentation

Patients with myocarditis can be asymptomatic or present with reduced exercise capacity, fatigue, and dyspnea. Around 60% of patients present with antecedent arthralgias, malaise, fever, sweats, or chills consistent with viral infections (e.g., diarrhea and/or vomiting with coxsackievirus, dyspnea with SARS-CoV-2) that they report occurred 1–2 weeks before the onset of symptoms (22, 25, 160). Patients may present with arrhythmias in the form of syncope, palpitations due to heart block, life-threatening bradyarrhythmias, and ventricular tachyarrhythmias or even sudden cardiac death, which is often associated with exertional exercise (22). Chest pain can range from mild to acute pain which is associated with myopericarditis in 35% of cases (18, 25). Severe cases of myocarditis can mimic myocardial infarction (161). Severe forms of myocarditis, such as fulminant myocarditis, can progress rapidly and result in acute myocardial failure and cardiogenic shock.

A recent study examined sex differences in clinical presentation of patients with myocarditis and found 82% (*n* = 63) were male, the mean age of patients was the same (around age 40), and BMI was similar at around 27 kg/m^2^ (162). Percent LVEF was similar in men (51 ± 13%) compared to women (57 ± 12%, *p* = 0.14). However, more men presented with symptoms of chest pain while women presented more often with dyspnea. Classic laboratory tests such as high sensitivity troponin T (hs-TnT), C-reactive protein (CRP), N-terminal-pro hormone-probrain natriuretic peptide (NT-proBNP), leukocytes, and thrombocytes were not found to display sex differences except for myoglobin and creatine kinase, with creatine kinase remaining significantly different after controlling for sex-specific reference ranges (162). Overall, comorbidities such as non-obstructive CAD, arterial hypertension (aHTN), hyperlipidemia, diabetes mellitus, atrial fibrillation, atrial flutter, and heart failure were rare and did not differ by sex (162). This is likely due to myocarditis occurring in young, relatively healthy patients. Larger studies are needed to better understand sex differences in the clinical presentation of myocarditis.

Several comorbidities increase the risk of heart failure including hypertension, diabetes, obesity, smoking history, and hyperlipidemia. Many of these factors increase risk more in women than men (163). Type 2 diabetes is an important risk factor for heart failure for both sexes, but data from the Framingham Heart Study found that diabetic women had a five-fold greater risk of heart failure while diabetic men had a two-fold greater risk (164). Similar findings have been reported for the risk of hypertension and heart failure (163). In another example, obesity is more prevalent in women than men and its association with heart failure risk is greater in women. However, heart failure in women is more closely associated with HFpEF, while risk of heart failure in myocarditis is associated with HFrEF (163, 165). A recent study of patients with HFpEF from three clinical trials (4,458 women and 4,010 men) found that women were older, more often obese and had hypertension while men were more likely to develop atrial fibrillation and CAD (166). Despite these risk factors, women had a lower risk of death compared to men (166). Importantly, all of these risk factors are higher in Black than White women and men (167), while myocarditis in the US occurs predominantly in White individuals. Thus, these factors are less likely to play an important role in the risk of developing myocarditis because it occurs in young individuals, prior to the clinical onset of many of these risk factors. Currently, the greatest known risk factors for myocarditis are young age and male sex, with the most frequent etiologic agents being viral infections worldwide and *Trypanosoma cruzi* in South America (Chagas disease).

Currently, we are not aware of any studies that examine the effect of gender on myocarditis and DCM outcomes based on their study design. These studies are needed in order to understand the contribution of gender to clinical sex differences outcomes.

## 8. Sex differences in biomarkers

Men are at an increased risk of developing heart failure from a number of CVDs including atherosclerosis, myocardial infarct, myocarditis, and DCM (168). Heart failure prevalence increases with advancing age in both genders, but increases dramatically in women >55 years of age in spite of better LV function than men (107, 169, 170). The strongest predictor of mortality in men is NYHA classification (107). Patients with chronic heart failure who have elevated levels of inflammation or inflammatory mediators/biomarkers have a worse prognosis (171). There are a number of traditional biomarkers that are used to diagnose myocarditis such as high sensitivity troponin T, troponin I, NT-proBNP, creatine kinase, and kappa or lambda immunoglobulin free light chains, but these markers are not disease specific (22, 162, 172). Tumor necrosis factor (TNF) and IL-6 are well known serum biomarkers of heart failure, along with IL-1β, but IL-1β is better as a tissue biomarker rather than a serum biomarker, as its sera levels are low. IL-1β contributes to Th17 responses by increasing the level of IL-6 where it combines with IL-23 and TGFβ-1 to drive Th17 immune responses while inhibiting regulatory T cells (Figure 1).

Over the past 10 years, a number of novel serum biomarkers have been reported that may improve the diagnosis of myocarditis and predict progression to cardiomyopathy/DCM and heart failure including sera soluble ST2 (sST2), myoglobin, IL-17-associated miRs, Th17-associated cytokines, and Th17 vs. Treg ratios (55, 142, 173–175). A study of around 300 patients with clinically suspected and/or biopsy confirmed myocarditis revealed that sST2 was elevated only in men under 50 years of age but not in women, and that higher sST2 levels were associated with elevated NYHA class heart failure (55). There was a trend for higher sST2 levels in women over 50 years of age with myocarditis, but the study was underpowered to confirm the relationship in women (55). Note that soluble sST2 that is a biomarker for heart failure is also known as interleukin-1 receptor-like 1 (IL1RL1 GeneID: 9173). This biomarker has been frequently confused, especially in the CVD literature, with the gene with the same abbreviation called suppression of tumorigenesis-2 (ST2 GeneID: 6761), which is found on chromosome 11 and represents a putative locus associated with cancer. Another study found an association between increased serum sST2 levels and fulminant myocarditis, a severe form of myocarditis that requires rapid detection to prevent a fatal outcome (176). However, only four patients were examined and so sex differences were not reported. Recently, sera myoglobin levels were found to be a strong predictor of acute myocarditis based on cardiac MRI and this sera biomarker was also detected in a viral model of myocarditis (174). Myoglobin was found to be significantly increased in men with myocarditis compared to women (*p* = 0.04) (162). In that study, there were no sex difference in hs-TnT, CRP, or NT-proBNP (162). Th17 cells and their associated cytokines (i.e., IL-17A, IL-6, IL-23) were found to directly correlate with heart failure in a study of patients with clinically suspected myocarditis/acute DCM (142). Importantly, IL-17A levels were found to be increased in males compared to females. Men were also found to have an association between a polymorphism in IL-17, rs763780, and an increased risk of DCM (142). This association of IL-17/Th17-associated cytokines with myocarditis was recently confirmed by separate investigators. They went on to identify a Th17-associated microRNA that was detected in the serum of patients and mice with myocarditis (autoimmune viral model) but not in patients or mouse models of myocardial infarct (175). The group did not report whether sex differences exist in the biomarker, but that the microRNA retained its diagnostic value in models after adjustment for age, sex, ejection fraction, and serum troponin level (175). Future studies should examine whether sex and age differences exist in these biomarkers which may provide insight into their role in the pathogenesis of disease.

## 9. Sex and gender differences in management

Standard treatment strategies for myocarditis and DCM remain as guideline-based heart failure therapies. There is only limited available literature on whether sex differences exist in the use of pharmacologic therapies for myocarditis or DCM. The Intervention in Myocarditis and Acute Cardiomyopathy-2 (IMAC-2) trial, which included 373 patients (38% women), found no significant difference in the use of angiotensin receptor blockers (ARBs), angiotensin converting enzyme (ACE) inhibitors or beta-blockers between men and women (37).

Most trials and meta-analyses suggest that medications used for the treatment of HFrEF reduce event rates in women. However, individual and sex-specific differences in drug absorption, distribution, metabolism, and excretion could affect drug doses needed for optimal efficacy and safety in patients with myocarditis and DCM (112, 177–180). Although HFrEF guidelines have a gender neutral dose recommendation for medications, women typically have a lower body weight, higher proportion of body fat, and lower plasma volume than men (181). This might result in higher maximum plasma concentrations of ARBs, ACE inhibitors, and beta-blockers in women compared to men using a similar dose. A *post hoc* analysis by Santema et al., reported sex-based differences in clinical outcomes in patients with HFrEF (182). In the Biostat-HF and ASIAN-HF registries, women treated with ACE inhibitors, ARBs, and beta-blockers had approximately 30% lower risk of death or hospitalization for heart failure at 50% of the guideline recommended doses (182). Women had no further benefit at higher doses. These data suggest that a lower target dose based on sex might be more appropriate (182). This is important because women with heart failure experience twice higher rates of adverse events (e.g., ACE inhibitor cough) from medication compared to men (180, 183, 184).

Importantly, fewer women compose clinical heart failure trials that guidelines are based on (185). Although it can be viewed that fewer women are recruited to trials, the sex difference in composition in trials may simply reflect sex differences in disease indicating that trials need to go longer to increase the number of women enrolled in the study. Challenges to this include the increased cost, yet it may be necessary to improve our understanding of sex and gender differences in disease. One important outcome of the underrepresentation of women is that a lack of evidence leads to women being less likely to be prescribed certain evidence-based medications; and when these medications are prescribed for women, dosing tends to be suboptimal (186, 187). It has been reported that male physicians use lower drug dosages and fewer drugs in female patients (188). In contrast, female physicians were found to have superior communication skills leading to better therapy adherence and improved outcomes in their patients as a consequence (189). Additionally, male patients seem to be less therapy adherent than female patients (190). These differences reflect a complex combination of sex and gender effects on the efficacy of available therapies.

### 9.1. Pharmacologic therapy

#### 9.1.1. Beta-blockers

While it has been established that biologic sex influences the function of the autonomic nervous system, clinical trials investigating beta-blockers have not sufficiently addressed this fact (191). Most beta-blocker trials were conducted at a time when investigators did not appreciate the effect of sex/sex hormones on pharmacokinetics. No studies to our knowledge have specifically examined the effect of beta-blockers on myocarditis or examined sex differences in their effect.

In a study of sex differences in congestive heart failure outcomes after using bisoprolol or metoprolol, both β1-selective β-adrenergic antagonists, Simon et al. found that women had improved survival for all factors examined but that the survival benefit in women was regardless of beta-blocker therapy (192). In contrast, a study conducting *post hoc* analysis of data in women with heart failure (*n* = 898) found that metoprolol improved outcomes in women similar to men (193), which was confirmed later by a meta-analysis of three clinical trials examining the effect of beta-blockers on heart failure (194). There is a need for studies to be conducted with the current understanding of the importance of sex differences to determine whether conclusions from these older studies are correct.

#### 9.1.2. ACE inhibitors

The influence of estrogen on the renin-angiotensin-aldosterone-system (RAAS) has been discussed in numerous publications, but its clinical implications remain unknown (191, 195–198). Over the years a number of clinical trials have shown the beneficial effects of ACE inhibitors in HFrEF patients without considering whether sex differences exist (199–202). However, several studies have examined sex differences. Garg et al. showed a 3% higher survival for men compared to women who received ACE inhibitor therapy in a meta-analysis of approximately 7,000 patients (23% women) enrolled in 32 randomized trials (203). The ATLAS trial compared low and high doses of lisinopril (2.5–5 mg daily vs. 32.5–35 mg daily) in 3,164 patients (648 women) with NYHA class II to IV chronic heart failure and LVEF ≤30% in a double blind randomized controlled trial (204). Patients receiving a high dose of lisinopril had a lower risk of death or hospitalization, which benefited men more than women. Finally, a meta-analysis of the six largest ACE inhibitor trials also found a significant survival benefit for men, but not for women (205). These data indicate that the benefit of ACE inhibitors is greater in men than women.

#### 9.1.3. Angiotensin receptor blockers

Patients with systolic heart failure who cannot tolerate ACE inhibitor therapy are sometimes treated with ARBs. In the Effects of High-dose vs. Low-dose Losartan on Clinical Outcomes in Patients with Heart Failure (HEAAL) study, the authors performed a randomized, double-blind trial in 255 sites with 3,846 heart failure patients (30% women) with a NYHA class II-IV, LVEF ≤40% and an ACE inhibitor intolerance (206). Patients were allocated into low vs. high dose groups (50 mg vs. 150 mg) and their all-cause mortality was compared. The higher dose was more beneficial for men, while the outcome for women did not differ between the two dose levels (206). A population study comparing sex differences in therapy response of ARBs vs. ACE inhibitors in 19,698 patients (10,223 women) found that women seem to benefit more from ARBs than men (207). These findings correlate with those of other authors (208, 209).

#### 9.1.4. Angiotensin receptor-neprilysin inhibition

Neprilysin is a membrane bound endopeptidase that cleaves and degrades vasoactive peptides, including natriuretic peptides, bradykinin, and adrenomedullin. Neprilysin inhibition has been evaluated to counteract neurohormonal overactivation in the treatment of heart failure. However, neprilysin also breaks down angiotensin II, so there is limited efficacy with lone neprilysin inhibition. The combination of ACE inhibitor therapy and neprilysin inhibition was associated with increased angioedema (210). Angiotensin receptor-neprilysin inhibition (ARNI), in the form of sacubitril-valsartan, was compared to enalapril in the Prospective Comparison of (ARNI) with Angiotensin Converting Enzyme Inhibitor (ACEI) to Determine Impact on Global Mortality and Morbidity in Heart Failure (PARADIGM-HF) trial (211). ARNI therapy was associated with a reduction in the risk of heart failure hospitalization and death, and there is now a class I indication for ARNI therapy in the treatment of patients with HFrEF (46). In the PARADIGM-HF trial, only 21.8% of patients enrolled were women. However, ARNI therapy was shown to reduce cardiovascular mortality and heart failure hospitalizations in both men and women (HR 0.80, 90.72–0.90; HR 0.77, 0.62–0.95, respectively; *p* = 0.63) (212). A previous prospective registry in 10 centers examined sex differences in efficacy, safety, and tolerability of sacubitril-valsartan and found no difference in discontinuation of ARNI therapy, no difference in received dose, and no difference in adverse events between women and men. A greater proportion of women did have an improved functional class, and female sex was considered an independent predictor of functional class improvement (213). In a *post hoc* analysis of patients with HFrEF enrolled in the Prospective Study of Biomarkers, Symptom Improvement and Ventricular Remodeling During Entresto Therapy for Heart Failure (PROVE-HF) trial, sex differences in biomarkers, health status, and remodeling endpoints were evaluated. This analysis demonstrated a reduction in NT-proBNP and improvement in cardiac remodeling parameters after initiation of ARNI therapy in both men and women. Women experienced improvement in cardiac remodeling parameters earlier, and women also experienced greater improvement in perceived quality of life based on changes in their Kansas City Cardiomyopathy questionnaire. This reiterated the benefit of ARNI therapy for both sexes.

Angiotensin receptor-neprilysin inhibition therapy has a class IIb indication for treatment of heart failure with preserved ejection fraction. This recommendation was based on the Prospective Comparison of ARNI with ARB Global Outcomes in Heart Failure with Preserved Ejection Fraction (PARAGON-HF) trial in which the effects of sacubitril-valsartan were compared with valsartan (214). Of the participants analyzed, 52% were women, which is one of the largest populations of women studied in a HFpEF trial. This trial did not meet its primary endpoint, identified as the composite of first and recurrent heart failure hospitalizations and cardiovascular death. A separate subgroup analysis found a more favorable treatment effect in women compared with men, with sacubitril-valsartan leading to a greater reduction in HF hospitalizations. Further analysis demonstrated that women enrolled in the trial were older, more obese, and had a lower median NT-proBNP and lower estimated glomerular filtration rate compared with men, highlighting the difference in clinical profile between women and men with HFpEF.

#### 9.1.5. Sodium-glucose cotransporter-2 inhibitors

Sodium-glucose cotransporter-2 inhibitors (SGLT2i) have been shown to reduce HF hospitalizations and cardiovascular death across all ranges of LVEF, and there is now a class I indication for SGLT2 inhibitors in HFrEF and a class IIa indication for HFpEF (46). SGLT2 inhibitors block glucose absorption at the proximal renal tubule. Additional mechanisms of action making them beneficial in heart failure are speculative, including reduction in fibrosis and potential improvement in myocardial metabolism and endothelial function. Recent trials, including Dapagliflozin and Prevention of Adverse Outcomes in Heart Failure (DAPA-HF) (215) and the Empagliflozin Cardiovascular Outcome Event Trial in Type 2 Diabetes Mellitus (EMPA-REG) (216), demonstrated a disproportionately lower enrollment of women compared with men with 23.4 and 29%, respectively, similar to other HFrEF trials. A prespecified subgroup analysis of DAPA-HF evaluated sex differences in drug efficacy. This study found that dapagliflozin reduced the risk of worsening heart failure and improved symptoms and quality of life similarly between men and women. This prespecified subgroup analysis also highlighted the different clinical profile between men and women with HFrEF, with women being older, less likely to have an ischemic etiology, with worse renal function, and with lower rates of HF hospitalizations and cardiovascular death compared to men. The effects of dapagliflozin on estimated glomerular filtration rate, body weight, and systolic blood pressure appeared to be similar between men and women. Safety data and tolerability for dapagliflozin in women were reassuring, with no increased adverse events specifically in women (217).

#### 9.1.6. Aldosterone antagonists

As discussed earlier, sex may influence the effect of drugs on the RAAS. Similar to ACE inhibitors and ARBs, there is no major study on aldosterone antagonists examining sex differences. Women have physiologically higher aldosterone levels, whilst aldosterone is known as a stimulator for proinflammatory pathways, cardiac remodeling, and the development of epicardial adipose tissue which is a driver of HFpEF pathogenesis (218–221). This indicates that inhibiting the final common path of RAAS might be more beneficial for women compared to men (218–220, 222). The Treatment Of Preserved Cardiac function in heart failure with an Aldosterone antagonist Trial (TOPCAT) was a randomized, double-blinded trial that compared spironolactone vs. placebo in 3,445 patients with HFpEF. In a *post hoc* analysis of the study population, Shah et al. found women to benefit from spironolactone across the whole LVEF spectrum compared to men who had a benefit only at lower LVEF (183, 223). All-cause mortality was also reduced by 34% in women, with no advantage for men (223). Another *post hoc* analysis performed by Merrill et al. confirmed the possibility of sex specific all-cause mortality in patients treated with spironolactone (224). This effect was only shown in all-cause mortality and not cardiovascular mortality alone, which the authors attribute to the lack of statistical power (224).

#### 9.1.7. Digoxin

The Digitalis Investigation Group trial showed that digoxin reduces hospitalization for heart failure but has no effect on mortality (225). Several *post hoc* subgroup analyses of the Digitalis Intervention Group trial population created controversy regarding digoxin therapy in women. Rathore et al. showed that all-cause mortality in HFrEF patients treated with digitalis was 5.8% higher in women (95% CI 0.5–11.1) (226). In another retrospective study including 1,926 women from the Digitalis Investigation Group trial population, digoxin treatment was found to be a significant independent covariate predicting all-cause mortality (227). Adams et al. performed a continuous multivariable analysis on 4,944 HFrEF patients of the Digitalis Investigation Group trial population, showing a significant linear relationship between serum digoxin concentration and mortality but no differences between the sexes (228).

### 9.2. Devices

#### 9.2.1. Implantable cardiac defibrillator

The risk of sudden cardiac death remans higher in men than women at all age groups (229), and develops in women about 10 years later than in men. Women are underrepresented in randomized implantable cardiac defibrillator (ICD) trials and are 40% less likely to obtain ICD therapy than men (230, 231). A meta-analysis of five ICD clinical trials found no significant difference in overall mortality between women and men, but women experienced significantly fewer appropriate ICD interventions (232). Importantly, women have a higher chance of complications during and after device implantation (233, 234). A recent study of 4,506 heart failure patients (76% male) found that women with ICDs had significantly less first and recurrent life-threatening ventricular arrhythmias than men, especially in patients with non-ischemic cardiomyopathy, suggesting that this therapy may be less effective for women (235).

#### 9.2.2. Cardiac resynchronization therapy

Cardiac resynchronization therapy-defibrillator (CRT-D) implantation is an established therapy for patients with chronic heart failure and a broad QRS complex. In a meta-analysis of pooled individual patient level data from three CRT-D trials, women with a relatively narrow QRS width between 130 and 149 ms benefited from CRT-D more than men. In this group, women had a 76% reduction in heart failure or death (absolute CRT-D to ICD difference, 23%; HR 0.24, 95% CI 0.11–0.53; *p* < 0.001) (236, 237). Women who receive CRT therapy show a therapy response of 90% over a wide range of QRS duration (130–175 ms) (238). Women with left bundle branch block and CRT do have significantly higher ventricular tachycardia-free survival than men, as shown in a multicenter retrospective study in 460 patients (105 women) of the Incidence of Arrhythmia in Spanish Population With a Medtronic Implantable Cardiac Defibrillator Implant national registry (UMBRELLA) (239). All-cause mortality did not differ between sexes in this study (239). Because females have such impressive benefits from CRT, improved screening and advocacy for CRT implantation in women should be considered (240).

#### 9.2.3. Mechanical circulatory support

Continuous flow left ventricular assist devices (LVAD) are utilized as bridge to transplant therapy (BTT) or destination therapy in chronic end-stage heart failure as well as patients with acute heart failure with hemodynamic compromise and cardiogenic shock, including patients with myocarditis. No large-scale clinical trials have investigated the use of these devices specifically in patients with myocarditis. One study of ventricular assist devices (VADs) used in 11 patients (6 women) with acute viral myocarditis showed similar survival among women and men, but more men required reoperation and more women developed right heart failure (241). A review of the use of VADs as a bridge to transplant in 6 patients (2 women) with giant cell myocarditis reported that 3 men and 1 woman were still alive after transplant, whereas 1 woman died of an embolic stroke and 1 man died of a hemorrhagic stroke before transplant (21).

Although mechanical circulatory support has increased for both women and men, particularly in the current era of continuous flow LVADs, information on sex and gender differences remains limited because sex-specific results are infrequently reported. Continuous flow LVADs have replaced the prior generation of pulsatile flow LVADs. Prior studies have also demonstrated that women in the pulsatile flow era had increased risk of mortality, with female sex being an independent predictor for hemorrhagic and ischemic stroke (242). More recent studies did not demonstrate sex differences in neurologic outcomes, and in the current continuous flow era, female sex does not appear to correlate with increased risk of mortality (243).

A previous study that systematically compared outcomes in women and men using LVADs as a bridge to transplant reported no survival difference between the sexes, but fewer women than men underwent heart transplantation (244). In the study, more women (72 of 104, 69%) than men (184 of 362, 51%) had non-ischemic cardiomyopathy. Adverse event rates were similar between women and men except for hemorrhagic stroke, which occurred more frequently in women, and device-related infections, which occurred more frequently in men (244). DeFilippis et al. recently described a United Network for Organ Sharing (UNOS) sample in BTT LVAD recipients, and women were found to have an increased risk of waitlist mortality (245). Maukel et al. found that in patients with LVADs implanted as destination therapy, women had increased rates of device replacement and recovery compared with men, but men and women did not differ in clinical outcomes including death or transplant (246). The HeartMate 3 LVAD is the only currently commercially available durable continuous-flow LVAD after a recall of the HeartWare device. In MOMENTUM 3 (Multicenter Study of MagLev Technology in Patients Undergoing Mechanical Circulatory Support Therapy with HeartMate 3), HeartMate 3 was found to be superior to the prior generation axial continuous-flow LVAD (HeartMate II) in the primary endpoint of survival free of disabling stroke or reoperation to replace or remove the device. There was no sex difference in stroke risk, but it is important to note that the HeartMate 3 cohort only consisted of 31 women (247).

An evaluation of all LVAD-related Emergency Department visits from 2010 to 2018 found significant sex differences. 27% of LVAD-related emergency department visits were female patients while only 21% of all patients with LVAD support in the study were female, indicating that women were seeking care in the Emergency Department more frequently than men. Importantly, women were found to have a lower likelihood of hospital admission compared with men despite similar presentation and were less likely to undergo cardiac catheterization or additional testing, highlighting inequities in their treatment (248).

### 9.3. Transplant

Cardiac transplantation is reserved for patients who are refractory to optimal medical therapy and mechanical circulatory support. Men are more frequently referred for heart transplantation than women in both the US and Europe. In a recent retrospective analysis of patients referred for advanced heart failure therapies, only 26.6% were women (249). In an assessment by the International Society for Heart and Lung Transplantation, only 23% of patients who underwent heart transplantation between 2005 and 2010 were women (250). A study that examined DCM specifically found an even greater gender imbalance in referral for heart transplantation of 1:6 at the German Heart Center Berlin and 1:5 in the Eurotransplant database (251). The authors concluded that the sex difference imbalance seemed to be due to referral bias, as women in both transplantation cohorts had more severe heart failure but fewer relative contraindications than men at the time of referral. There is no data on whether there are sex differences in referral for transplantation for patients with myocarditis. In October 2018, the UNOS implemented a new donor heart allocation system, and recent analyses have highlighted new trends in the allocation system, such as increased temporary mechanical circulatory support (252). Further studies are needed to determine the impact on sex differences in outcomes with the new allocation system.

## 10. Future directions

There are a number of areas that are important to consider for the future. Disaggregation of sex-based analyses is a critical part of studies that include both sexes (8). Not reporting sex-based analyses can give an incorrect depiction of a disease or treatment as sex-neutral in risk or effect. This is particularly important for studies involving the immune response, where hormone response elements often alter gene responses in opposite directions (i.e., estrogen response element decreases genes while androgen response element increases the same genes) so if the analysis is not conducted according to sex then the two sexes cancel each other out to suggest no relationship. Even among cardiovascular clinical trials that include both sexes, a minority (33%) were found to actually report the data analysis (253). Future work needs to include data, even if non-significant, between the sexes (1). Although many advances have been made in the past decade, many of the same gaps remain. There is a need for a better understanding of the causes of myocarditis in order to identify more specific biomarkers of disease onset and progression. This can be better accomplished using translational models of myocarditis and by obtaining cardiac biopsies. There remains a need to understand the effect of genes that are expressed on the X chromosome in mediating sex differences in the immune response during myocarditis. There is also a need to bank serum and tissue samples of myocarditis and DCM patients in the US, as has been done for years overseas, so that a better understanding of the viruses and other causes of myocarditis can be identified, and disease-specific therapies developed. Myocarditis differs profoundly by sex, and data in animal and human studies need to interpret their findings in relation to sex and gender differences to improve care for myocarditis and DCM patients in the future. In order to gain an understanding of how gender affects myocarditis and DCM, studies need to be designed specifically to examine this question.

## 11. Conclusion

Over the last decade, interest in myocarditis and DCM has dramatically increased and many new insights have been gained. Once a limited topic on what has been considered a rare condition, myocarditis has entered the public eye with the outbreak of the worldwide SARS-CoV-2 pandemic. While the past decade of research has brought tremendous insight into the pathogenesis of myocarditis and DCM, many of the same problems identified a decade ago remain. There are no unified global standards for the diagnosis of myocarditis. Most of the clinical insights on myocarditis have come from biopsy samples, which are rarely taken in the US and especially for the most common form of myocarditis, lymphocytic myocarditis, which hinders progress. There are few biobanks of myocarditis samples in the US which stymies evidence-based research advances. Even with the increased understanding of the importance of sex and gender, there is lack of analysis of sex and age differences in most studies, both clinically and in animal models. Furthermore, there is a lack of studies that specifically examine the role of gender on disease pathogenesis or outcomes. Importantly, older studies need to be reassessed considering our current understanding of the effects of sex and age on myocarditis and DCM in all facets of the disease, from epidemiology and pathogenesis, to current treatment guidelines and therapies. Fortunately, highly translational animal models of myocarditis that progress to DCM exist and are increasing our understanding of the role of sex and age in disease. In conclusion, a heightened understanding of sex and gender differences is critical for improving diagnostic strategies and clinical management that will lead to optimal sex- and gender-based care for patients with myocarditis and DCM.

## Author contributions

DF and DB: conceptualization and writing—original draft. DF: project administration. DF, DB, NM, BH, ML, LC, and KB: writing—review and editing. All authors have read and agreed to the published version of the manuscript.
